# The impact of COVID-19 pandemic on the treatment of acute appendicitis in China

**DOI:** 10.1007/s00384-021-04031-4

**Published:** 2021-10-13

**Authors:** ZhiXue Zheng, Jing Tao Bi, Ya Qi Liu, Xuan Cai

**Affiliations:** Department of General Surgery, Beijing Jishuitan Hospital, 31 Xinjiekou East Street, Xicheng District, Beijing, 100035 China

**Keywords:** Coronavirus disease 2019 (COVID-19), Acute appendicitis, Treatment

## Abstract

**Objective:**

This research aims to analyze the impact of the novel coronavirus pandemic on the hospital visits of patients with acute appendicitis.

**Methods:**

The retrospective analysis was designed to look at the treatment of acute appendicitis in the Department of General Surgery in Beijing Jishuitan Hospital before and during the COVID-19 pandemic (2019–2020). Data was analyzed by the numbers of patients, sex, age, onset time, fever or not, laboratory examination, imaging test, and treatment. And we analyzed the differences between the “pre-COVID group” and “during-COVID group”.

**Results:**

Compared with the year 2019, the number of acute appendicitis patients has diminished substantially during the COVID-19 pandemic (2020), but the number elevated with the control of the pandemic. Even if we did not find the differences of the treatment before and during the pandemic (*P* = 0.932), the onset time to emergency was significantly longer (*P* < 0.001), and more patients had showed fever (*P* < 0.001) during the COVID-19 pandemic. And the total number of white blood cells and C reactive protein level were significantly higher in 2020 than those in 2019 (*P* = 0.006, 0.003). And the same result was found in patients with appendiceal fecalith (*P* = 0.047).

**Conclusion:**

During the pandemic of the new coronavirus pneumonia, the number of patients with acute appendix treatment dropped significantly, mainly because it took longer than before, and the condition was more severe. It can be seen that the new coronary pneumonia has a great impact on the patients’ medical treatment behavior, and the active prevention and treatment of the new coronavirus pneumonia is currently an important and urgent issue.

## Introduction

At the end of December 2019, an unexplained pneumonia caused by a novel coronavirus infection was found in some hospitals in Wuhan, Hubei Province, China. After virus typing detection and identification by scientists, the virus was named the Coronavirus disease 2019 (COVID-19) by WHO. Since then, this virus has been detected in many countries worldwide and has now threatened global human health [1, 2]. Subsequently, the Law of the People’s Republic of China on the Prevention and Treatment of Infectious Diseases clearly specified that COVID-19 is a Class B infectious disease, and the prevention and control measures of Class A infectious diseases should be taken, among which isolation policies are an important part [3–5]. Since the COVID-19 pandemic began to circulate, people’s lives, work, and psychology have changed significantly, especially having a great impact on people’s medical behavior. For national or regional isolation policies during pandemics, some researchers have reported that the number of emergency visits in hospitals has decreased significantly compared with before, and the same is true for patients undergoing emergency surgery.

Acute appendicitis is one of the most common acute abdominal diseases in emergency surgery [6], especially during the pandemic of the new coronavirus, patients with this disease have undergone major changes compared to the previous treatment [7]. Based on this situation, this research discussed the treatment of patients with newly diagnosed acute appendicitis in our hospital from January 2019 to December 2020, and compared the treatment methods of patients before and after the timeline of the new coronavirus pandemic to observe the influence of the pandemic on the treatment behavior of patients.

## Methods

### Physical data

In this research, a total of 1135 patients with acute appendicitis with complete data of emergency visits to the Department of Surgery of Beijing Jishuitan Hospital from January 2019 to December 2020 were included. In 2019, 652 patients were treated, including 314 females and 338 males. In 2020, 483 patients were treated, including 221 females and 262 males. They were divided into two groups according to the time period of consultation, the pre-pandemic group of patients who visited the clinic from January 2019 to December 2019, and the inter-pandemic group from January 2020 to December 2020.

### Research design

The main purpose of this study is to compare and analyze the following information, including the gender, age, time from onset to consultation, blood routine, C-reactive protein, fever, imaging examination, and treatment methods of the patients included in the study. The same applies to the number of visits, visit time, examination results, and treatment options for patients with acute appendicitis during the pandemic.

### Statistical analysis

SPSS 20.0 statistical software was used for data analysis. Enumeration data were analyzed by chi-square *χ*^2^ test. Measurement data were expressed as mean ± standard deviation and analyzed by *t*-test. *P* < 0.05 was considered statistically significant.

## Results

### Comparison of patient visits before and during the COVID-19 pandemic

**Fig. 1:**
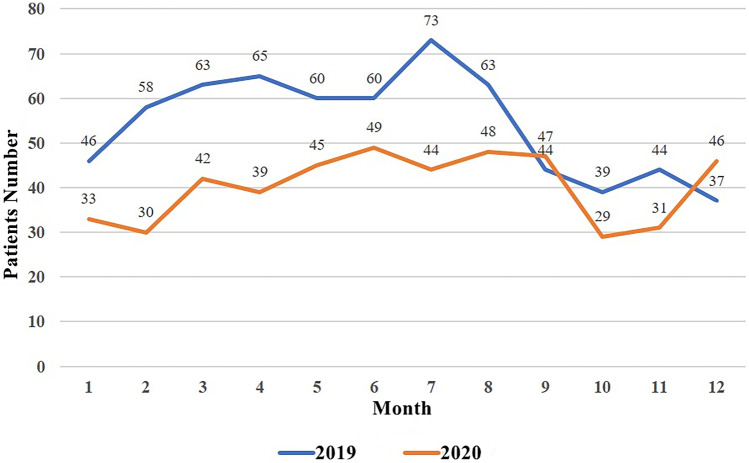
Comparison of the number of patients with acute appendix in 2019 and 2020

A total of 652 patients with acute appendicitis were included in 2019 in the pre-pandemic group and 483 patients in 2020 in the group during the coronavirus disease pandemic. The overall number of patients decreased significantly compared with that before the pandemic, by about 25.9% (169/652). And it was reduced by 33.6% (143/425) in the first 8 months. The monthly incidence in 2020 was significantly lower than the number of patients attending the hospital during the same period before the pandemic, and only the number in December 2020 was higher than that of patients attending the hospital in December 2019, including a significant decrease in patients attending the hospital during the pandemic outbreak in Wuhan (January–April 2020) and during the pandemic in the new place of Beijing (June–August 2020). With the improvement of the outbreak of the Coronavirus disease, the number of patients presenting with acute appendicitis gradually increased (Fig. 1).

There were no significant differences in the male to female ratio and the mean age between patients with acute appendicitis who visited the group during the pandemic (2020) and the group before the pandemic (2019) (*P* = 0.422, 0.120). The time from onset to consultation was significantly longer in the group of patients during the pandemic than in the group of patients before the pandemic, and the proportion of patients who visited the hospital with onset more than 24 h was significantly higher in 2020 than in 2019, and the difference between the two groups was statistically significant (*P* < 0.001). In terms of treatment options, there was no significant difference between the two groups of patients (*P* = 0.932). The patients in the group during the pandemic (2020) also had significantly more fever at consultation than those in the group before the pandemic (2019) (*P* < 0.001), and the difference was statistically significant (Table 1).

**Table 1:** Diagnosis and treatment of patients before and after the pandemic

Years	Number of visits in 2019	Number of visits in 2020	*P*
Gender			0.422
Female	314 (48.2%)	221 (45.8%)	
Male	338 (51.8%)	262 (54.2%)	
Age (years)	37.17 ± 0.58	38.59 ± 0.71	0.120
Onset time			< 0.001
< 24 h	574 (88.0%)	354 (73.3%)	
≥ 24 h	78 (12.0%)	129 (26.7%)	
Treatment mode			0.932
Medication	517 (79.3%)	384 (79.5%)	
Surgical treatment	135 (20.7%)	99 (20.5%)	
Fever or not			< 0.001
None	627 (96.2%)	418 (86.5%)	
Yes	25 (3.8%)	65 (13.5%)	

### Comparison of examination results of patients before and during the pandemic

The mean value of the total white blood cell count examined in the laboratory of patients with acute appendicitis in the group during the pandemic (2020) was significantly higher than that of patients in the group before the pandemic (2019), and the difference between them was statistically significant (12.69 ± 0.19 vs. 12.03 ± 0.15, *P* = 0.006), while there was no significant difference in the proportion of neutrophils between the two groups (*P* = 0.223). C-reactive protein levels were significantly higher in patients who visited the clinic in 2020 than patients who visited the clinic in 2019, and the difference was statistically significant (25.14 ± 2.17 vs. 17.48 ± 1.38, *P* = 0.003). The proportion of patients with acute appendicitis with appendiceal fecalith found by abdominal CT or ultrasound was higher in 2020 than in 2019, and the difference was statistically significant (*P* = 0.047) (Table 2).

**Table 2:** Comparison of examination results of patients before and after the pandemic

Years	Number of visits in 2019		Number of visits in 2020	*P*
White blood cell count (× 10^9^/L)	12.03 ± 0.15	12.69 ± 0.19		0.006
Percentage of neutrophils	0.78 ± 0.00	0.79 ± 0.00		0.223
C-reactive protein (mg/L)	17.48 ± 1.38	25.14 ± 2.17		0.003
Appendiceal fecalith				0.047
None	516 (79.1%)	358 (74.1%)		
Yes	136 (20.9%)	125 (25.9%)		

## Discussion

The Coronavirus disease is caused by severe acute respiratory syndrome coronavirus type 2 (SARS-CoV-2). Since the end of 2019, the number of cases of novel coronavirus infection has continued to increase, and more than 150 million cumulative confirmed cases have been diagnosed worldwide [8–12]. Due to the infectivity of the Coronavirus disease, the high mortality rate of severe disease, and the lack of effective treatment, it is easy to make the public panic and fear, and has a significant impact on people’s psychology and medical behavior [13–16]. In the face of the pandemic situation of the Coronavirus disease, the Chinese government and health authorities actively take strict pandemic prevention measures to reduce population aggregation and close contact, and take strict isolation policies for confirmed cases, suspected cases, and close contacts, so that the pandemic situation in China can be gradually controlled, while the situation of the Coronavirus disease abroad is still severe [17].

Under the influence of disease pandemics and psychological panic among the population, people have different degrees of anxiety about seeking medical treatment, which increases the possibility of treatment delay, and the total number of emergency patients is significantly lower than before [18, 19]. Acute appendicitis is a common surgical emergency disease. This research analyzed the changes of patients’ hospitalization before and during the pandemic, and discussed the impact of the pandemic on patients. In this research, we found that during the pandemic period of novel coronavirus pneumonia, the number of patients with initial acute appendicitis who visited the hospital was reduced by about 1/4 compared with that before the pandemic, especially during the national pandemic and Beijing pandemic periods in the first 8 months. Moreover, the time from onset to consultation of patients during the pandemic was also significantly longer than that before the pandemic, the number of patients who presented more than 24 h was significantly increased, and at the same time, patients who experienced fever at consultation were also significantly more than those before the pandemic. The similar results were observed in other countries [20–22]. The reason for this may be that patients are concerned about being infected with COVID-19 during going to the hospital, and that quarantine measures in some communities also make it difficult for patients to visit the hospital. These reasons lead to patients not visiting a doctor in time when they have abdominal pain and other early symptoms of acute appendicitis, resulting in delayed treatment and fever, and they have to go to the hospital for diagnosis and treatment. In particular, some patients who experience fever due to severe disease are more likely to develop anxiety about COVID-19, which delays the time to consultation. This also further illustrates the reason why the white blood cell count and C-reactive protein of patients during the pandemic were significantly higher than those before the pandemic. The overall number of patients with acute appendicitis who chose surgical treatment during the pandemic was less than that before the pandemic, but the proportion of surgical patients in the overall patients was not significantly different from that before the pandemic. The reason is that during the pandemic period of the Coronavirus disease in China, the patients present late and have severe disease, but timely surgical treatment is a very important medical means. Therefore, although the overall number of patients attending the hospital has been reduced, the proportion of patients undergoing surgery has not been significantly reduced. In addition, due to the lack of awareness of the disease by patients, especially during the occurrence of the novel coronavirus outbreak, patients through various media (WeChat, Weibo, website, etc.) to get a lot of information about COVID-19 highly contagious, underestimate the severity of acute appendicitis, and miss the opportunity for early treatment. Therefore, the patient was already in a more severe condition at the time of treatment, but surgery was an important way to quickly relieve the symptoms. Finally, the cost of patients, the adjustment of hospital beds during the pandemic, and the choice of patients themselves are all important factors affecting the choice of treatment methods. In the late 2020, with the gradual control of COVID-19 in China and the gradual opening of communities and public places, people’s awareness of their own health has been improved, and they also actively seek medical treatment after symptoms appear. Therefore, the number of patients with acute appendicitis begins to increase in the second half of 2020. Although this research is based on COVID-19 cases for this kind of disease diagnosis and treatment of acute appendicitis, but it also reflects the outbreak from the side of the effects of medical behavior, especially for the emergency treatment of acute disease has had an impact, caused the delay of diagnosis and treatment of many patients, so as to make the part of the patient condition is more difficult to restore. Therefore, it is very necessary to actively control COVID-19 outbreak.

At present, China has started to fully vaccinate COVID-19 vaccine, which plays a positive role in preventing COVID-19, and people also begin to go to hospitals for treatment of diseases. At the same time, we should expand the publicity of disease and health education, improve health awareness, let people know that if they find that they are unwell, go to the doctor early, the treatment effect will be better. The weakness of this research lies in that it is only a single-center study in China, and it fails to involve and analyze the changes of patients’ medical treatment in other hospitals, which may lead to selective deviation. In addition, the results may be incomplete due to hospital bed issues, risk of infection, and differences in patient awareness, and choice of acute appendicitis disease. However, COVID-19 is an important factor affecting people’s behavior to seek appropriate medical care. The global pandemic has not been effectively controlled, and there are still local cases of infection in China, which has a great impact on all aspects of people’s lives. Only by actively vaccinating people, cutting off the transmission route, immediately reporting the discovery, and precise prevention and control can make people’s lives and medical treatment return to normal.

## Conclusion

During the pandemic of the new coronavirus pneumonia, the number of patients with acute appendicitis treatment dropped significantly, mainly because it took longer to visit the hospital than before, and the condition was more severe. It can be seen that the new coronary pneumonia has a great impact on the patients’ medical treatment behavior, and the active prevention and treatment of the new coronavirus pneumonia is currently an important and urgent issue.
